# The Effect of Cognitive Behavioral Intervention on Cognitive Situation, Quality of Life, Depression, Anxiety and Stress Levels of Menopausal Women: Mixed Method Study

**DOI:** 10.1192/j.eurpsy.2025.776

**Published:** 2025-08-26

**Authors:** F. Atkan, F. Oflaz

**Affiliations:** 1 Istinye University, Faculty of Health Sciences; 2 Koç University, School of Nursing, İstanbul, Türkiye

## Abstract

**Introduction:**

Menopause causes physiological, cognitive, and psychological changes in women and negatively affects women’s quality of life.

**Objectives:**

This study aimed to examine the effects of cognitive behavioral approach-based intervention on the cognitive situation, quality of life, anxiety, depression, and stress levels experienced by women after menopause with mixed-method research.

**Methods:**

The research was carried out between March 2022 and August 2023 as a mixed-method research consisting of three phases (quantitative, qualitative, and intervention). Eighty women (experiment=27, control=53) attended in the quantitative phase. Quantitative data were collected before & after intervention by The Sociodemographic Data Form, The Montreal Cognitive Assessment Test, The Menopause-Specific Quality of Life Scale, and The Depression Anxiety Stress Scale. In the intervention phase, a six-session, cognitive behavioral approach-based nursing intervention was conducted with five groups using online Zoom. Hermeneutic phenomenology design was used in the qualitative phase. Qualitative data were collected online, via Zoom platform, and through three focus group interviews. Qualitative data were evaluated by the Thematic Analysis method. In the analysis of quantitative data, descriptive statistics, Independent Samples Groups t-test, Mann-Whitney U Test, and Wilcoxon Test were used.

**Results:**

There was no statistically significant difference between intervention and control groups in terms of sociodemographic characteristics, age, age of onset of menstruation, and menopause. Post-intervention cognitive scores (Z=-3.936, p=0.001) and psychosocial quality of life scores (Z=-2.771, p=0.006) of women who were in the intervention were higher than their pretest scores. There was no statistically significant difference in the post-intervention mean scores between groups in terms of other variables (p>0.05). The themes were loss, stigma, loneliness, not being understood, aging, loss of health, sexuality, acceptance, self-awareness, and coping ability. Women’s perceptions of menopause changed mostly functionally after the intervention study.

**Conclusions:**

The research findings showed that Cognitive Behavioral Intervention had some curative effects on women’s cognitive changes and psychosocial changes they experienced during menopause. Nurses working with menopausal women can use Cognitive Behavioral approaches to manage the changes brought about by menopause effectively.

**Disclosure of Interest:**

None Declared

